# Prevalence, clinical characteristics, and long-term outcomes of new diabetes diagnosis in elderly patients undergoing percutaneous coronary intervention

**DOI:** 10.1038/s41598-024-65426-1

**Published:** 2024-06-27

**Authors:** Zheng-Kai Xue, Xin-Ya Dai, Jia-Yi Ren, Tong Liu, Yu-Kun Zhang, Su-Tao Hu, Peng Wang, Xue Wu, Jing-Kun Zhang, Gary Tse, Soohyung Park, Cheol-Ung Choi, Byoung-Geol Choi, Seung-Woon Rha, Kang-Yin Chen

**Affiliations:** 1 Tianjin Key Laboratory of Ionic-Molecular Function of Cardiovascular Disease, Department of Cardiology, Tianjin Institute of Cardiology, The Second Hospital of Tianjin Medical University, 23, Pingjiang Road, Hexi District, Tianjin, 300211 China; 2 Department of Cardiology, Tianjin Union Medical Center, Nankai University, Tianjin, 300121 China; 3 Institute for Global Health Sciences, University of California, San Francisco, CA USA; 4 Cardiovascular Research Institute, University of California, San Francisco, CA USA; 5 Cardiovascular Center, Korea University Guro Hospital, 148, Gurodong‑ro, Guro‑gu, Seoul, 08308 Republic of Korea; 6 Diabetes Research Unit, Cardiovascular Analytics Group, Power Health Research Institute, Hong Kong, China; 7 Kent and Medway Medical School, Canterbury, UK; 8 School of Nursing and Health Studies, Hong Kong Metropolitan University, Hong Kong, China

**Keywords:** Newly diagnosed diabetes, Elderly, Coronary artery disease, Percutaneous coronary intervention, Interventional cardiology, Type 2 diabetes, Risk factors

## Abstract

Previous studies have reported associations between newly diagnosed diabetes and poor outcomes after percutaneous coronary intervention (PCI), but there is limited data focusing on elderly patients (age ≥ 65). This study aimed to analyze the prevalence and clinical implications of newly diagnosed diabetes in elderly patients who underwent PCI. From 2004 to 2021, a total of 2456 elderly patients who underwent invasive PCI at Korea University Guro Hospital were prospectively enrolled and followed up for a median of five years. The primary endpoint was five-year major adverse cardiovascular events (MACE). Cox regression was used to evaluate whether newly diagnosed diabetes impacted on long-term clinical outcomes. Newly diagnosed diabetes was presented in approximately 8.1% to 10.9% of elderly patients who underwent PCI. Those who had a new diagnosis of diabetes had a higher risk of MACE than previously known diabetes (25.28% vs. 19.15%, *p* = 0.039). After adjusting for significant factors, newly diagnosed diabetes remained an independent predictor of MACE (HR [hazard ratio] 1.64, 95% confidence interval [CI] 1.24–2.17, *p* < 0.001), cardiac death (HR 2.15, 95% CI 1.29–3.59, *p* = 0.003) and repeat revascularization (HR 1.52, 95% CI 1.09–2.11, *p* = 0.013), but not for non-fatal myocardial infarction (HR 1.66, 95% CI 0.94–2.12, *p* = 0.081). Newly diagnosed diabetes was associated with an increased risk of 5-year MACE compared with non-diabetes and previously diagnosed diabetes in elderly patients underwent PCI. More attention should be given to those elderly newly diagnosed diabetes population.

## Introduction

Patients with coronary artery disease (CAD) who also have diabetes mellitus (DM) experience more cardiovascular events compared to non-diabetic patients^1,2^. Many studies have demonstrated a higher prevalence of DM in patients who underwent percutaneous coronary intervention^3–5^ (PCI) and those with DM were at a heightened risk of cardiovascular events^6,7^, such as restenosis^8^, stent thrombosis^9^, and higher rates of revascularization^10^. Furthermore, several researches have indicated that about 16–19% of patients undergoing PCI had newly diagnosed DM^4,5,11^. And newly diagnosed DM is shown to be associated with increased short-term and one-year major adverse cardiovascular and cerebral events (MACCE)^5,12^. However, there were limited data regarding the impact of newly diagnosed DM up to five years outcomes in elderly patients (age ≥ 65) who underwent PCI.

Thus, the objective of this study was to assess the prognostic implications of newly diagnosed DM in elderly patients undergoing PCI after a median follow-up of 5 years, in comparison to individuals with known DM or without DM.

## Methods

### Study population

Since 2004, all patients who underwent invasive PCI at Korea University Guro Hospital (KUGH) in Seoul, South Korea, were prospectively enrolled in the KUGH-PCI registry. It is a prospective study of all comers conducted at a single center that reflects ‘real world’ practice. All patients’ demographic data, clinical characteristics, description of PCI, medications on discharge, and blood tests including admission blood glucose, and glycated hemoglobin A1c (HbA1c) level were collected. The study exclusion criterion was defined as the exclusion of patients aged < 65 years, PCI procedural failure, no-drug-eluting stents (DES) or absent HbA1c level. For clinical diagnoses and clinical events, standard definitions were used for all variables related to patients and lesions. For all the participants or their legal guardians, a thorough literal and verbal explanation of study procedures was provided before their written informed consents were obtained. The Institutional Review Board of KUGH approved this study (#KUGH10045). All methods were performed in accordance with relevant guidelines and regulations.

### Study definitions

In this study, newly diagnosed DM was defined as admission HbA1c ≥ 6.5%, without known DM history or current use of hypoglycemic agents. Multivessel disease was defined as at least two coronary vessels or left main coronary disease with more than 50% luminal narrowing. Diffuse long lesions referred to lesion length > 30 mm. Chronic total occlusion (CTO) was an obstruction of a native coronary artery that lasts for more than three months. Target lesion revascularisation (TLR) referred to the repeat revascularization in the previously implanted stent or 5 mm proximal or distal to the stented segment. Target vessel revascularisation (TVR) referred to the intervention in the same treated vessel. And non-TVR referred to a revascularization of any segment of the non-target coronary artery.

The primary clinical outcomes of interest were major adverse cardiovascular events (MACE) defined as the composite of cardiac death, non-fatal myocardial infarction (MI), and any repeat revascularization (including TLR, TVR, and non-TVR) among the three groups. Secondary outcomes were the individual components of MACE, all-cause mortality, TLR, TVR, non-TVR, stent thrombosis and stroke.

### Statistical analysis

For continuous variables, data were expressed as mean ± standard deviations, or median (IQR) for variables that are not normally distributed. Differences among the three groups were evaluated by one-way ANOVA or Kruskal Wallis test and a post hoc test was applied: the Tukey’s Honestly Significant Difference test or Steel–Dwass test. For categorical variables, differences were expressed as counts and percentages and analyzed with χ^2^ or Fisher’s exact test between the groups as appropriate. Random forest method was used to impute missing values (using the randomForest package in the R language).

To adjust for potential confounders, univariable Cox proportional hazards model analysis was performed. We tested available 20 baseline clinical and angiographic variables that could be of relevance: sex, age, ST-segment elevation myocardial infarction (STEMI), cardiogenic shock, previous myocardial infarction (MI), previous PCI, previous coronary artery bypass grafting (CABG), hypertension, chronic kidney disease (CKD), atrial fibrillation (AF), smoking, multivessel disease, left main coronary lesion, bifurcation lesion, ca1cification lesion, diffuse long lesion, CTO and number of stents, total stent length. All variables with *P* < 0.05 or considered of clinical implications were included for further multivariable Cox analysis. A nomogram was constructed to visualize the risk factors for clinical outcomes with best discrimination and calibration. A two-tailed *p*-value of < 0.05 was considered to be statistically significant. The Bonferroni correction was used to adjust the *P* values for pairwise comparisons. Various 5-year clinical outcomes were estimated with Kaplan–Meier method, and differences between groups were compared with log-rank test. Data processing and analysis were performed using R version 4.3.0 (2023–04–21), along with Storm Statistical Platform (www.medsta.cn/software).

## Results

### Population

**Figure 1:**
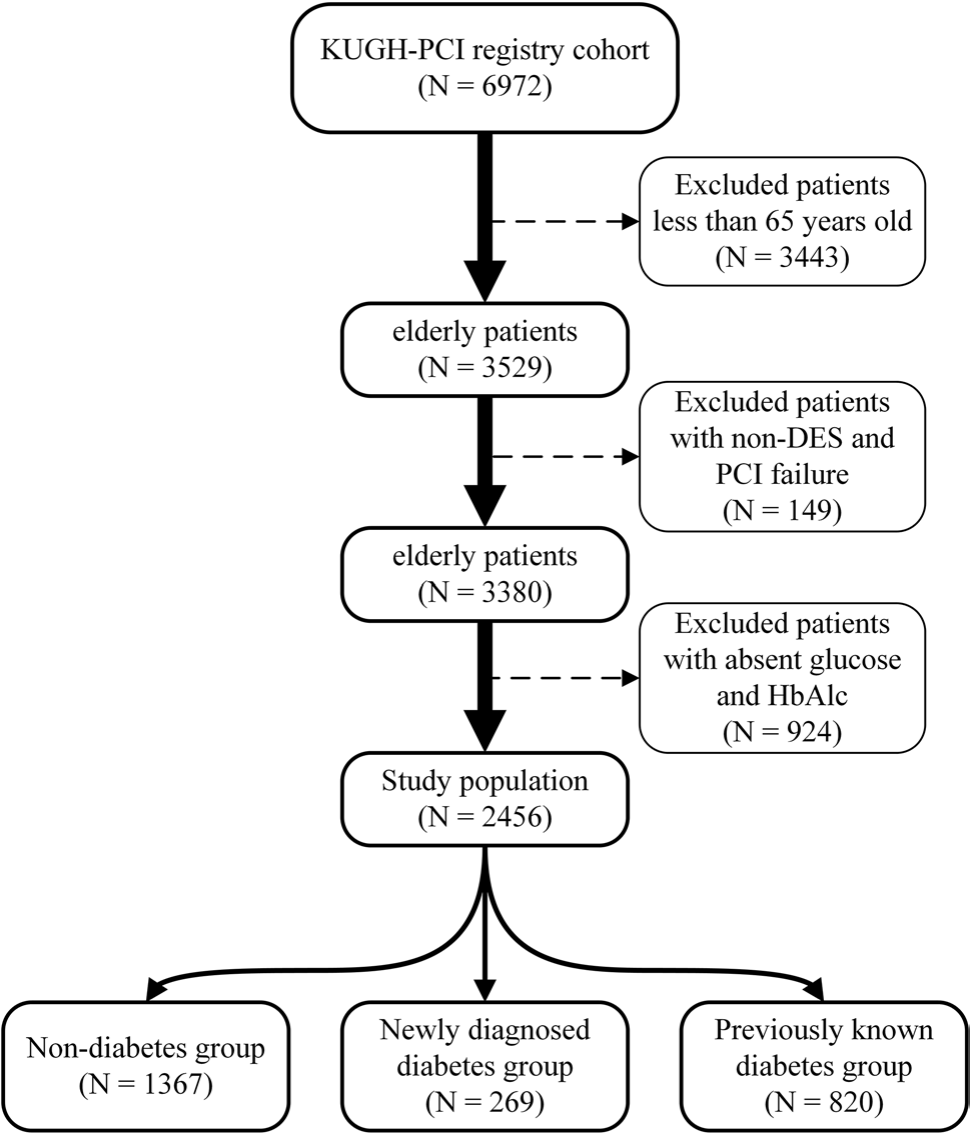
Flowchart of the study population and distribution of the population. DES = drug-eluting stents, HbA1c = hemoglobin A1c, KUGH = Korea University Guro Hospital, PCI = percutaneous coronary intervention.

A total of 6972 patients who underwent PCI were enrolled during January 2004 and June 2021 (Fig. 1). Of these, 3529 were elderly patients (≥ 65 years old), 287 (8.1%) patients were newly diagnosed with DM, 1127 (32.0%) patients had previously known DM, and 2115 (59.9%) patients did not have DM. The median follow-up was 1806 days (5.0 years, median 825–2740 days [2.3–7.6 years]). Eventually, patients aged < 65 years (n = 3443), PCI procedural failure or no-DES (n = 149), absent HbA1c level (n = 924) were excluded (Fig. 1).

Considering the potential impact of stent generation and PCI procedural failure on clinical outcomes, a total of 2456 patients with DES, successful PCI revascularization and available record of blood glucose and HbA1c level were included in the final analysis. In this study population, 269 (10.9%) patients were newly diagnosed DM, 820 (33.4%) patients had previously known DM, and 1367 (55.7%) patients were non-DM. The median follow-up was 1975 days (5.4 years, median 882–2925 days [2.4–8.0 years]).

### Baseline clinical and angiographic characteristics

The baseline clinical characteristics of patients categorized by glycemic status are detailed in Table 1. Newly diagnosed diabetic patients were younger (median age, 71 vs. 73 years old, *p* = 0.026), had higher rates of previous MI (2.23% vs. 0.24%, *p* = 0.005), previous PCI (4.46% vs. 0.00%, *p* < 0.001), previous CABG (1.86% vs. 0.24%, *p* = 0.007) and lower rates of hypertension (71.38% vs. 79.27%, *p* < 0.001), AF (3.35% vs. 7.07%, *p* = 0.004) and CKD (5.95% vs. 10.98%, *p* < 0.001) compared to those with previously known DM. Higher levels of HbA1c (median level, 7.1% vs. 7.0%, *p* < 0.001), total cholesterol (median level, 4.29 vs. 4.01 mmol/l, *p* < 0.001), total triglycerides (median level, 1.30 vs. 1.19 mmol/l, *p* < 0.001) and low-density lipoprotein cholesterol (median level, 2.79 mmol/l vs. 2.30 mmol/l, *p* < 0.001) were more common in newly diagnosed diabetes group and non-diabetes group compared to previously known diabetes group.

**Table 1:** Clinical characteristics and five-year outcomes of patients and comparison among groups.

Variable	Total (n = 2456)	Glycemic status			*P*
		Non-diabetes (n = 1367)	Newly diagnosed diabetes (n = 269)	Previously known diabetes (n = 820)	
Demographic characteristics					
Age, year, M (Q_1_, Q_3_)	72.00 (68.00–77.00)	72.00 (68.00–77.00)	71.00 (68.00–76.00)	73.00 (69.00–77.00)	0.026
Male, n (%)	1409 (57.37)	820 (59.99)	133 (49.44)	456 (55.61)	0.003
ACS, n (%)	1822 (74.19)	1023 (74.84)	193 (71.75)	606 (73.90)	0.557
STEMI, n (%)	442 (18)	273 (19.97)	42 (15.61)	127 (15.49)	0.017
Cardiogenic shock, n (%)	92 (3.75)	40 (2.93)	10 (3.72)	42 (5.12)	0.032
Previous MI, n (%)	14 (0.57)	6 (0.44)	6 (2.23)	2 (0.24)	0.005
Previous PCI, n (%)	29 (1.18)	17 (1.24)	12 (4.46)	0 (0.00)	< 0.001
Previous CABG, n (%)	11 (0.45)	4 (0.29)	5 (1.86)	2 (0.24)	0.007
Hypertension, n (%)	1744 (71.01)	902 (65.98)	192 (71.38)	650 (79.27)	< 0.001
Smoking, n (%)	490 (19.95)	287 (20.99)	57 (21.19)	146 (17.80)	0.169
CKD, n (%)	152 (6.19)	46 (3.37)	16 (5.95)	90 (10.98)	< 0.001
Atrial fibrillation, n (%)	124 (5.05)	57 (4.17)	9 (3.35)	58 (7.07)	0.004
Laboratory data					
WBC, × 10^9^/l, M (Q_1_, Q_3_)	7.40 (5.90–9.40)	7.10 (5.70–9.20)	7.30 (6.10–9.10)	7.80 (6.30–9.90)	< 0.001
Platelet, × 10^9^/l, M (Q_1_, Q_3_)	216 (180–257)	215 (181–255)	222 (182–260)	214 (176–262)	0.366
Hb, g/l, M (Q_1_, Q_3_)	128 (116–139)	131 (119–142)	127 (115–137)	124 (111–136)	< 0.001
HbA1c, %, M (Q_1_, Q_3_)	6.00 (5.60–6.90)	5.70 (5.50–6.00)	7.10 (6.70–8.00)	7.00 (6.40–7.90)	< 0.001
Glucose, mmol/l, M (Q_1_, Q_3_)	6.89 (5.67–9.28)	6.33 (5.44–7.67)	8.00 (6.22–10.67)	8.39 (6.17–11.68)	< 0.001
Creatine, mmol/l, M (Q_1_, Q_3_)	76.0 (61.9–93.7)	73.4 (61.9–88.4)	79.6 (68.1–97.2)	78.7 (62.8–103)	< 0.001
TC, mmol/l, M (Q_1_, Q_3_)	4.27 (3.59–0.57)	4.42 (3.70–5.20)	4.29 (3.62–5.22)	4.01 (3.36–4.78)	< 0.001
TG, mmol/l, M (Q_1_, Q_3_)	1.20 (0.82–1.69)	1.16 (0.79–1.64)	1.30 (0.97–1.90)	1.19 (0.86–1.77)	< 0.001
HDLc, mmol/l, M (Q_1_, Q_3_)	1.11 (0.93–1.32)	1.14 (0.96–1.37)	1.06 (0.93–1.29)	1.06 (0.88–1.27)	< 0.001
LDLc, mmol/l, M (Q_1_, Q_3_)	2.61 (1.97–3.31)	2.74 (2.12–3.41)	2.79 (2.02–3.49)	2.30 (1.73–2.97)	< 0.001
Angiographic characteristics and stent information					
Multi-vessel disease, n (%)	758 (30.86)	398 (29.11)	80 (29.74)	280 (34.15)	0.044
Left main disease, n (%)	119 (4.85)	65 (4.75)	12 (4.46)	42 (5.12)	0.884
Bifurcation lesion, n (%)	774 (31.51)	453 (33.14)	94 (34.94)	227 (27.68)	0.013
Calcification lesion, n (%)	465 (18.93)	226 (16.53)	60 (22.30)	179 (21.83)	0.003
Diffuse long lesion, n (%)	1104 (44.95)	559 (40.89)	147 (54.65)	398 (48.54)	< 0.001
Chronic total occlusion, n (%)	186 (7.57)	97 (7.10)	16 (5.95)	73 (8.90)	0.171
Number of stents, n, M (Q_1_, Q_3_)	1.00 (1.00–2.00)	1.00 (1.00–2.00)	1.00 (1.00–2.00)	1.00 (1.00–2.00)	< 0.001
Total stent length, mm, M (Q_1_, Q_3_)	32.00 (20.00–52.00)	30.00 (19.00–48.00)	33.00 (24.00–54.00)	34.00 (20.00–56.00)	< 0.001
Five-year major adverse cardiovascular events					
MACE, n (%)	436 (17.75)	211 (15.44)	68 (25.28)	157 (19.15)	< 0.001
Cardiac death, n (%)	125 (5.09)	53 (3.88)	21 (7.81)	51 (6.22)	0.005
No-fatal myocardial infarction, n (%)	87 (3.54)	49 (3.58)	16 (5.95)	22 (2.68)	0.042
Repeat revascularisation, n (%)	324 (13.19)	164 (12.00)	46 (17.10)	114 (13.90)	0.059
TLR, n (%)	161 (6.56)	88 (6.44)	23 (8.55)	50 (6.10)	0.357
TVR, n (%)	212 (8.63)	110 (8.05)	32 (11.90)	70 (8.54)	0.120
Non-TVR, n (%)	154 (6.27)	78 (5.71)	18 (6.69)	58 (7.07)	0.423
Total death, n (%)	247 (10.06)	110 (8.05)	36 (13.38)	101 (12.32)	< 0.001
Stent thrombosis, n (%)	41 (1.67)	23 (1.68)	5 (1.86)	13 (1.59)	0.946
Stroke, n (%)	112 (4.56)	61 (4.46)	8 (2.97)	43 (5.24)	0.291

ACS, Acute coronary syndrome; CABG, coronary artery bypass grafting; CKD, chronic kidney disease; Hb, hemoglobin; HbA1c = hemoglobin A1c; HDLc, high-density lipoprotein cholesterol; LDLc, low-density lipoprotein cholesterol. MACE, major adverse cardiovascular events, defined as the composite of cardiac death, no-fatal myocardial infarction, and revascularisation; MI, myocardial infarction; PCI, percutaneous coronary intervention; STEMI, ST-segment elevation myocardial infarction; TC, total cholesterol; TG, total triglycerides; TLR, target lesion revascularisation; TVR, target vessel revascularisation; WBC, white blood cell.

The angiographic characteristics and stent information across three groups are displayed in Table 1. Newly diagnosed DM and previously known DM patients had similar prevalence of multi-vessel disease (29.74% vs. 34.15%, *p* = 0.363), left main coronary disease (4.46% vs. 5.12%, *p* = 0.900), bifurcation lesions (34.94% vs. 27.69%, *p* = 0.067), ca1cification lesion (22.30% vs. 21.38%, *p* = 0.984), diffuse long lesions (54.65% vs. 48.54%, *p* = 0.185), and CTO (5.95% vs. 8.90%, *p* = 0.250). Thus, newly diagnosed DM and previously known DM patients had similar number of stents (1.69 ± 0.97 vs. 1.85 ± 1.11, *p* = 0.073) and total stent length (42.79 ± 27.10 mm vs. 43.70 ± 30.72 mm, *p* = 0.430). Over 98% of the patients was prescribed aspirin and clopidogrel after discharge. And there was no significant disparity between the utilization of antiplatelet therapy in all three groups (*p* > 0.100).

### Five-year clinical outcomes

The newly diagnosed and previously known DM groups exhibited higher rates of total death (13.38% vs. 8.05%, *p* = 0.011; 12.32% vs. 8.05%, *p* = 0.004), cardiac death (7.81% vs. 3.88%, *p* = 0.022; 6.22% vs. 3.88%, *p* = 0.025) and MACE (25.28% vs. 15.44%, *p* < 0.001; 19.15% vs. 15.44%, *p* = 0.039) compared to the non-diabetic group (Table 1). Newly diagnosed DM seemed to have higher risk of MACE (25.28% vs. 19.15%, *p* = 0.039) and a trend towards higher MI (5.95% vs. 2.68%, *p* = 0.058) than previously known DM. However, there were no statistically significant differences in terms of TLR, TVR, non-TVR, and stent thrombosis across the three groups.

**Figure 2:**
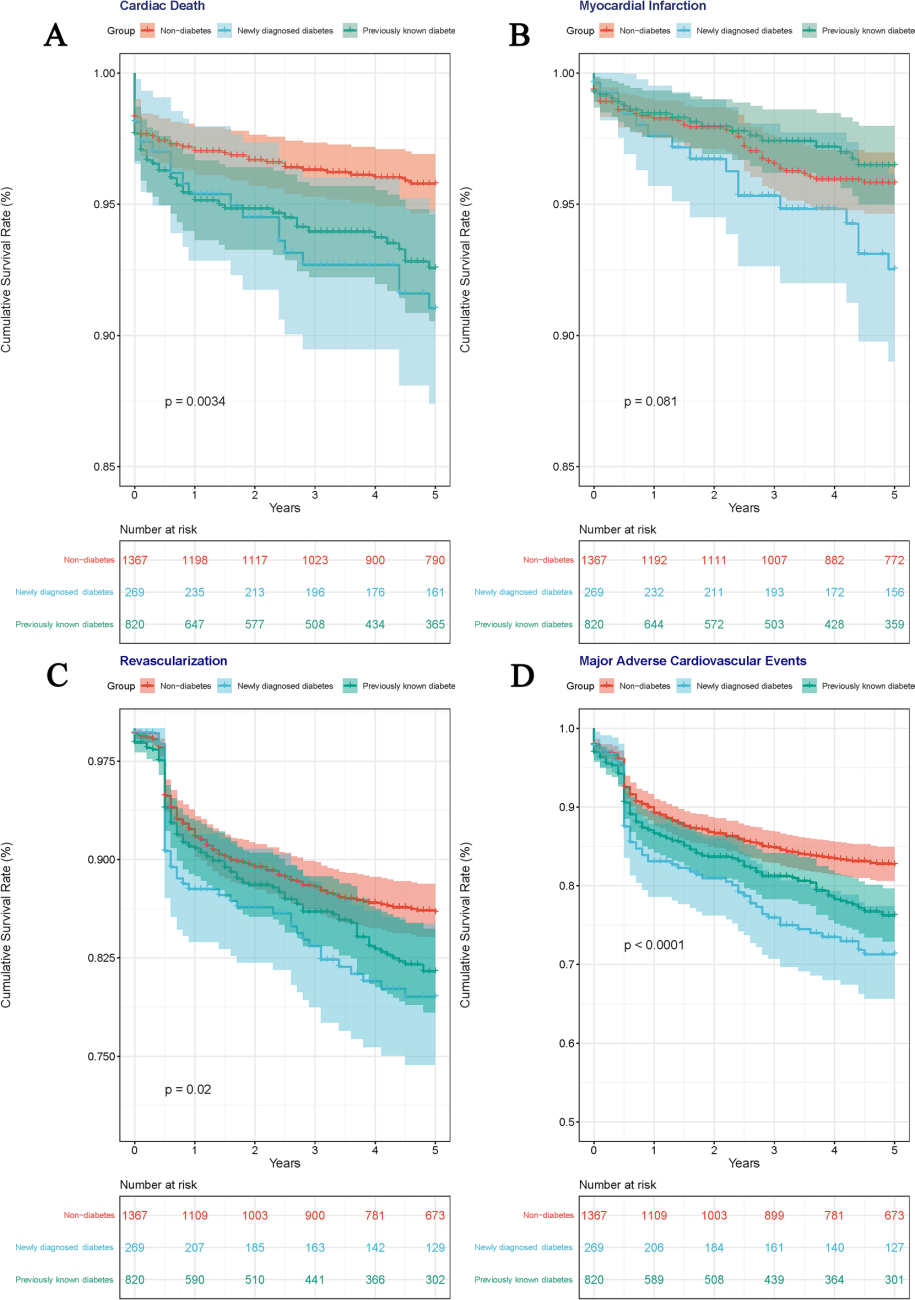
Kaplan–Meier survival curves for five-year cardiac death (**A**), no-fatal myocardial infarction (**B**), repeat revascularization (**C**) and major adverse cardiovascular events (**D**). Newly detected diabetes (blue), previously known diabetes (green), and non-diabetes (red).

Kaplan–Meier survival analysis demonstrated that the 5-year cumulative incidences of MACE were significantly higher in the newly diagnosed diabetes and previously known diabetes, compared to non-diabetic group (pairwise log-rank *p* < 0.001; pairwise log-rank *p* = 0.003; Fig. 2D), but the difference between newly diagnosed and previously known diabetes was not significant (pairwise log-rank *p* = 0.135). The components of MACE in different glycemic status are shown in Fig. 2A–C. The 5-year cumulative incidences of cardiac death and repeat revascularization were similar as MACE. Only 5-year cumulative incidence of no-fatal MI in newly diagnosed diabetes was significantly higher than that in previously known diabetes (pairwise log-rank *p* = 0.031).

### Multivariable Cox regression model

The detailed results could be found in Table 2 and more univariate Cox analysis information in Supplementary Material. Multivariate Cox regression showed that newly diagnosed diabetes was an independent predictor of MACE [*hazard ratio* (HR) 1.64, 95% confidence internal (CI) 1.24–2.17, *p* < 0.001], cardiac death (HR 2.15, 95% CI 1.29–3.59, *p* = 0.003) and repeat revascularization (HR 1.52, 95% CI 1.09–2.11, *p* = 0.013), except for the non-fatal MI (HR 1.66, 95% CI 0.94–2.12, *p* = 0.081). While previously known DM was only independently associated with five-year MACE (HR 1.24, 95% CI 1.01–1.54, *p* = 0.044). Besides, cardiogenic shock was the strongest predictor of MACE (HR 4.54, 95% CI 3.28–6.30, *p* < 0.001), cardiac death (HR 9.85, 95%CI 6.41–15.14, *p* < 0.001), non-fatal MI (HR 3.66, 95% CI 1.68–7.97, *p* = 0.001) and repeat revascularization (HR 1.88, 95%CI 1.07–3.29, *p* = 0.027). Additionally, CKD remained a significant predictor of MACE (HR 1.71, 95% CI 1.23–2.37, *p* = 0.001), cardiac death (HR 3.24, 95% CI 1.99–5.28, *p* < 0.001), and non-fatal MI (HR 2.24, 95% CI 1.11–4.51, *p* = 0.024).

**Table 2:** Independent predictors of five-year clinical outcomes after multivariable Cox adjustment for baseline clinical and angiographic characteristics.

Variables	HR (95%CI)	*P*-value	C-index (95% CI)
MACE (cardiac death, no-fatal myocardial infarction, or repeat revascularization)			
Glycemic status			0.627 (0.598–0.656)
Non-diabetes	Reference		
Newly diagnosed diabetes	1.64 (1.24–2.17)	< 0.001	
Previously known diabetes	1.24 (1.01–1.54)	0.044	
ST segment elevation myocardial infarction	1.41 (1.11–1.78)	0.005	
Cardiogenic shock	4.54 (3.28–6.30)	< 0.001	
Chronic kidney disease	1.71 (1.23–2.37)	0.001	
Left main coronary lesion	1.59 (1.07–2.35)	0.021	
Number of stents	1.25 (1.12–1.38)	< 0.001	
Cardiac death			
Age	1.06 (1.03–1.09)	< 0.001	0.796 (0.756–0.836)
Glycemic status			
Non-diabetes	Reference		
Newly diagnosed diabetes	2.15 (1.29–3.59)	0.003	
Previously known diabetes	1.44 (0.97–2.13)	0.068	
ST segment elevation myocardial infarction	2.25 (1.53–3.32)	< 0.001	
Cardiogenic shock	9.85 (6.41–15.14)	< 0.001	
Chronic kidney disease	3.24 (1.99–5.28)	< 0.001	
Left main coronary lesion	2.60 (1.56–4.34)	< 0.001	
No-fatal myocardial infarction			
Age	1.04 (1.01–1.07)	0.035	0.616 (0.551–0.681)
Glycemic status			
Non-diabetes	Reference		
Newly diagnosed diabetes	1.66 (0.94–2.92)	0.081	
Previously known diabetes	0.75 (0.45–1.25)	0.271	
Cardiogenic shock	3.66 (1.68–7.97)	0.001	
Chronic kidney disease	2.24 (1.11–4.51)	0.024	
Repeat revascularization			
Male	1.28 (1.02–1.62)	0.035	0.589 (0.557–0.621)
Glycemic status			
Non-diabetes	Reference		
Newly diagnosed diabetes	1.52 (1.09–2.11)	0.013	
Previously known diabetes	1.24 (0.97–1.59)	0.082	
Cardiogenic shock	1.88 (1.07–3.29)	0.027	
Number of stents	1.19 (1.08–1.30)	< 0.001	

MACE, Major adverse cardiovascular events, defined as the composite of cardiac death, no-fatal myocardial infarction, and revascularisation. The detailed covariates included in these models were listed in the "Methods" section. Covariates not statistically significant were not shown in the table.

**Figure 3:**
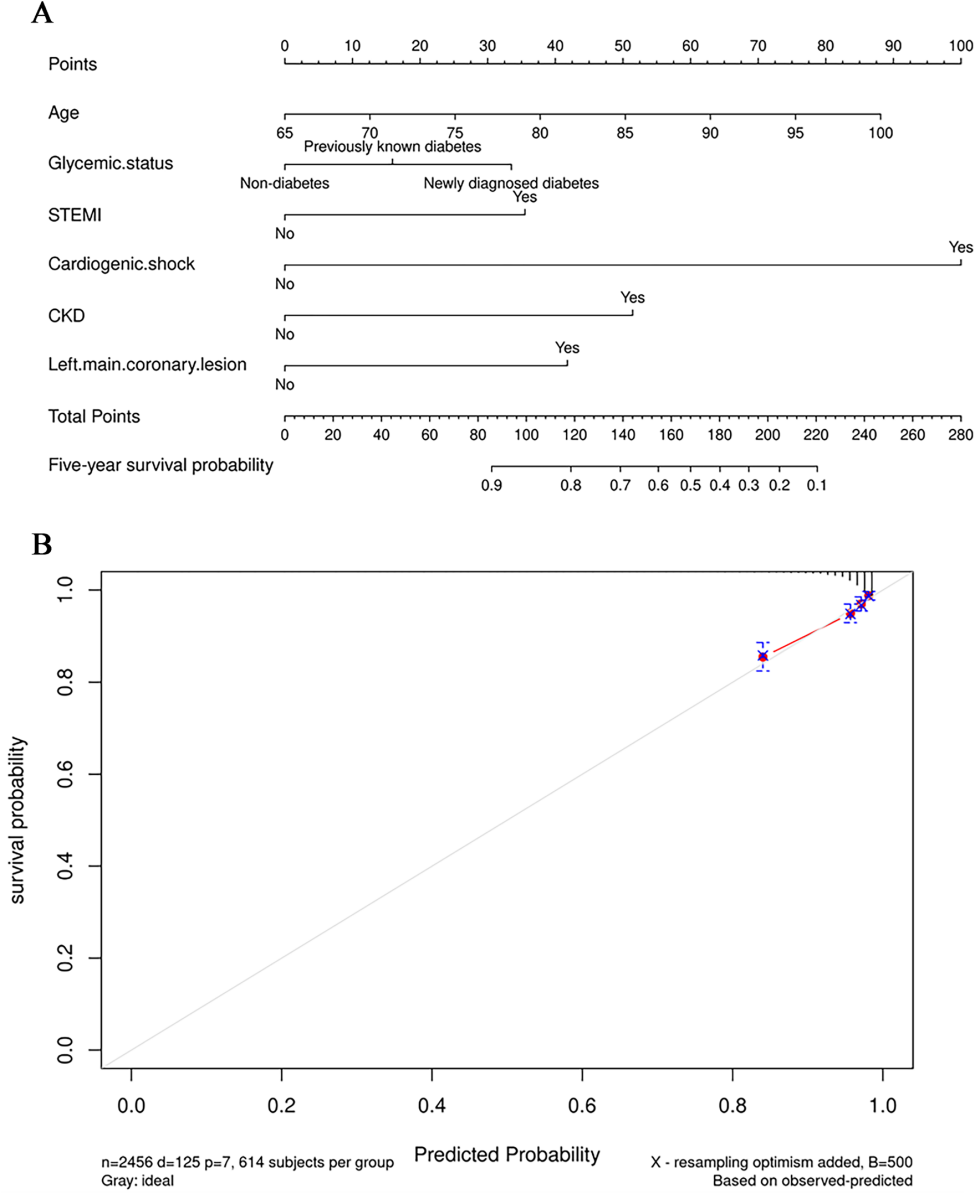
The figure (**A**) is a nomogram predicting the risk of five-year cardiac death in elderly patients underwent PCI. A score was given to the value of each variable on the point scale axis. By adding each score, a total score could be calculated and projected to the lower total score scale, so we could estimate five-year cardiac-death free survival probability. The figure (**B)** is the calibration curves for the nomogram. The x-axis represents the nomogram-predicted probability and y-axis represents the actual probability of cardiac death. Perfect prediction would correspond to the 45° gray dashed line. The red dotted line represents the entire cohort (n = 2456), and the blue solid line is bias-corrected by bootstrapping (B = 500 repetitions), indicating observed nomogram performance. CKD = chronic kidney disease, STEMI = ST-segment elevation myocardial infarction.

To assess the performance of the models described above, concordance index (C-index) was calculated, and calibration curve was determined for the best regression model. The multivariable Cox regression model for five-year cardiac death had the best discrimination with C-index of 0.796 (95% CI 0.756–0.836). The subsequent nomogram and calibration curve are showed in Fig. 3.

## Discussion

In this study, newly diagnosed DM was presented in approximately 8.1–10.9% of elderly patients who underwent PCI. Moreover, newly diagnosed DM was independently associated with increased risk of five-year MACE, and was a stronger risk factor compared with previously known DM.

Several studies have analysed the prevalence of newly diagnosed DM in specific CAD patients undergoing PCI and subsequent clinical outcomes. De la Hera et al.^4^ reported 16.2% of studied patients (stable angina or NSTEMI, n = 580) who underwent PCI was newly detected diabetes, based on the oral glucose tolerance teat (OGTT) after discharge. And newly diagnosed DM was not an independent predictor of 1-year outcomes. Tailakh et al.^5^ demonstrated that 19% of total 1151 CAD patients had newly diagnosed DM based on the HbA1c level. Newly diagnosed DM was independently associated with increased MACCE at one-month but not at one-year. Subgroup analysis showed male and patients younger than 75 years appeared higher one-year MACCE risk. Jimenez-Navarro et al.^13^ discovered that 21.4% of 374 CAD patients who underwent PCI were newly detected diabetes and previously known diabetes remained the only independent predictor of cardiovascular events in a mean follow-up of 35.8 ± 13.43 months. Tsuchida et al.^14^ reported 17% of the 298 patients studied had newly diagnosed DM, which was not a predictor of up to 10-year cardiovascular disease risk. Other studies focused on only STEMI patients^15,16^. Therefore, newly diagnosed DM seemed to have greater short-term impact and less long-term impact according to the above studies. But there is limited data to evaluate the impact of newly diagnosed DM in elderly patients.

As far as we know, our study focused specifically on elderly population and for the first time, investigating whether or not newly diagnosed DM impacted on five-year clinical outcomes in elderly patients undergoing PCI. The prevalence of newly diagnosed DM in elderly patients undergoing PCI was similar to that in prior studies across all age groups. All patients with DM, no matter newly or previously diagnosed, had more cardiovascular events than patients without DM, which has been investigated in numerous studies^12,17–19^. Our results are in agreement with previous findings, newly diagnosed diabetic patients were younger and had less comorbidities^5,12^. Furthermore, we reported more detailed information about angiographic characteristics of newly diagnosed diabetes than former studies. Despite having similar baseline to patients without DM, patients with newly diagnosed diabetes had comparable complicated coronary lesions with previous DM patients. Notably, newly diagnosed diabetic patients had more history of previous MI, PCI and CABG, along with higher HbA1c level, which may account for its poor prognosis^5,19–21^. A possible reason is that those patients with newly diagnosed DM may have been unaware of asymptomatic and uncontrolled DM for a long time. A recent study^22^ has shown similar impact of DM on increased risk of 5-year cardiovascular outcomes after PCI.

In the present study, we focused on elderly patients with newly diagnosed diabetes, a distinctive cohort that warrants special attention. It might be assumed that newly diagnosed diabetes has a lesser impact on elderly individuals. However, it is essential to recognize that older adults often face a unique set of challenges, including a higher risk of physical and mental frailty ^23,24^, a vulnerable immune system^25^, an increased burden of diabetes-related complications^26^, poorer treatment adherence^27^, and a potentially more conservative approach to blood glucose control due to concerns about hypoglycemia^28^. As a result, these factors may collectively contribute to the elevated incidence of adverse outcomes in the elderly population, which underscores the importance of our study's focus on this particular demographic. By acknowledging these factors and their implications, we can better appreciate why there is a need for a more targeted and nuanced approach to managing diabetes in elderly patients.

There are some limitations should be considered. Firstly, this was a single-center study, it is important to evaluate the findings in a larger population involving multiple centers. Despite a monocenter study, we included more than two thousand elderly patients underwent PCI to preliminarily explore the long-term outcomes of newly diagnosed DM. Secondly, there was no information available to evaluate the status of diabetes controlled, the treatment modalities, changes on medication details and adherence, which may affect the outcomes of patients undergoing PCI. There should be more specific work on those elements in the future. Thirdly, due to the absence of available variables, a mount of cases were excluded from the analysis, which could have biased our findings. Besides, it is possible that multivariable models may contain incomplete adjustments or unknown confounders that have not been consolidated.

## Conclusions

In conclusion, our study indicated that newly diagnosed DM was associated with an increased risk of five-year MACE compared with non-DM and previously diagnosed DM in elderly patients who underwent PCI. Our findings support the importance of identifying and treating diabetes at early stage for elderly CAD patients undergoing PCI. And it is necessary to monitor on the blood glucose, and perform HbA1c screening among high-risk elderly patients who undergoing PCI. It is hoped that more attention and care should be given to the older population.

### Supplementary Information

Supplementary Information.

## Data Availability

Data are available by contacting the corresponding author on reasonable requests.

## References

[1] Rawshani A, Rawshani A, Gudbjornsdottir S (2017). Mortality and cardiovascular disease in type 1 and type 2 diabetes. N. Engl. J. Med..

[2] Billinger M, Beutler J, Taghetchian KR (2008). Two-year clinical outcome after implantation of sirolimus-eluting and paclitaxel-eluting stents in diabetic patients. Eur. Heart J..

[3] Tada T, Kimura T, Morimoto T (2011). Comparison of three-year clinical outcomes after sirolimus-eluting stent implantation among insulin-treated diabetic, non-insulin-treated diabetic, and non-diabetic patients from j-Cypher registry. Am. J. Cardiol..

[4] de la Hera JM, Delgado E, Hernandez E (2009). Prevalence and outcome of newly detected diabetes in patients who undergo percutaneous coronary intervention. Eur. Heart J..

[5] Tailakh MA, Friger M, Zahger D (2017). Prospective study of the impact of diabetes mellitus newly diagnosed by glycated hemoglobin on outcomes in patients undergoing percutaneous coronary intervention. Eur. J. Intern Med..

[6] Mathew V, Gersh BJ, Williams BA (2004). Outcomes in patients with diabetes mellitus undergoing percutaneous coronary intervention in the current era: A report from the Prevention of REStenosis with Tranilast and its Outcomes (PRESTO) trial. Circulation.

[7] Wilson SR, Vakili BA, Sherman W (2004). Effect of diabetes on long-term mortality following contemporary percutaneous coronary intervention: Analysis of 4284 cases. Diabetes Care.

[8] Kornowski R, Mintz GS, Kent KM (1997). Increased restenosis in diabetes mellitus after coronary interventions is due to exaggerated intimal hyperplasia: A serial intravascular ultrasound study. Circulation.

[9] Iakovou I, Schmidt T, Bonizzoni E (2005). Incidence, predictors, and outcome of thrombosis after successful implantation of drug-eluting stents. JAMA.

[10] Sato T, Ono T, Morimoto Y (2012). Differences in clinical and angiographic outcomes with different drug-eluting stents in Japanese patients with and without diabetes mellitus. J. Cardiol..

[11] Muhlestein JB, Anderson JL, Horne BD (2003). Effect of fasting glucose levels on mortality rate in patients with and without diabetes mellitus and coronary artery disease undergoing percutaneous coronary intervention. Am. Heart J..

[12] Aguilar D, Solomon SD, Kober L (2004). Newly diagnosed and previously known diabetes mellitus and 1-year outcomes of acute myocardial infarction: The VALsartan In Acute myocardial iNfarcTion (VALIANT) trial. Circulation.

[13] Jimenez-Navarro MF, Fernandez-Pastor J, Garrido-Sanchez L (2015). Newly impaired glucose metabolism and prognosis after percutaneous revascularization. Cardiol. J..

[14] Tsuchida K, Mitsuma W, Sato Y (2020). Impaired glucose tolerance and future cardiovascular risk after coronary revascularization: A 10-year follow-up report. Acta Diabetol..

[15] Aggarwal B, Shah GK, Randhawa M (2016). Utility of glycated hemoglobin for assessment of glucose metabolism in patients with ST-segment elevation myocardial infarction. Am. J. Cardiol..

[16] Ostrominski JW, Vaduganathan M, Girish MP (2022). Missed opportunities for screening and management of Dysglycemia among patients presenting with acute myocardial infarction in North India: The prospective NORIN STEMI registry. Glob. Heart.

[17] Marso SP, Mercado N, Maehara A (2012). Plaque composition and clinical outcomes in acute coronary syndrome patients with metabolic syndrome or diabetes. JACC Cardiovasc. Imaging.

[18] Ojeda S, Pan M, Martin P (2014). Immediate results and long-term clinical outcome of patients with unprotected distal left main restenosis: The CORPAL registry (Cordoba and Las Palmas). JACC Cardiovasc. Interv..

[19] Selvin E, Steffes MW, Zhu H (2010). Glycated hemoglobin, diabetes, and cardiovascular risk in nondiabetic adults. N. Engl. J. Med..

[20] Baber U, Azzalini L, Masoomi R (2021). Hemoglobin A(1c) and cardiovascular outcomes following percutaneous coronary intervention: Insights from a large single-center registry. JACC Cardiovasc. Interv..

[21] Timmer JR, Hoekstra M, Nijsten MW (2011). Prognostic value of admission glycosylated hemoglobin and glucose in nondiabetic patients with ST-segment-elevation myocardial infarction treated with percutaneous coronary intervention. Circulation.

[22] Kosmidou I, Leon MB, Zhang Y (2020). Long-term outcomes in women and men following percutaneous coronary intervention. J. Am. Coll. Cardiol..

[23] Veronese N, Custodero C, Cella A (2021). Prevalence of multidimensional frailty and pre-frailty in older people in different settings: A systematic review and meta-analysis. Ageing Res. Rev..

[24] Soysal P, Veronese N, Thompson T (2017). Relationship between depression and frailty in older adults: A systematic review and meta-analysis. Ageing Res. Rev..

[25] Eggersdorfer M, Berger MM, Calder PC (2022). Perspective: Role of micronutrients and Omega-3 long-chain polyunsaturated fatty acids for immune outcomes of relevance to infections in older adults—A narrative review and call for action. Adv. Nutr..

[26] Gopalraj R (2017). The older adult with diabetes and the busy clinicians. Primary Care: Clin. Off. Pract..

[27] Chiatti C, Sustacchini S, Furneri G (2012). The economic burden of inappropriate drug prescribing, lack of adherence and compliance, adverse drug events in older people a systematic review. Drug Saf..

[28] Bruce DG, Davis WA, Davis TME (2018). Glycaemic control and mortality in older people with type 2 diabetes: The Fremantle Diabetes Study Phase II. Diabetes, Obesity and Metabolism.

