# Supplementary material for: Prevalence, clinical characteristics, and long-term outcomes of new diabetes diagnosis in elderly patients undergoing percutaneous coronary intervention
Source: Sci Rep. 2024 Jun 27;14:14814. doi: 10.1038/s41598-024-65426-1 (PMC11211319; doi:10.1038/s41598-024-65426-1)
Supplement: Supplementary file 1 — Supplementary Information. [file 41598_2024_65426_MOESM1_ESM.docx]

# Supplementary Material

**Univariate and multivariate Cox analysis for MACE**

| **Variables** | **Beta** | **S.E** | **Z** | **P** | **HR (95%CI)** | **m_Beta** | **m_S.E** | **m_Z** | **aP** | **aHR (95%CI)** |
| --- | --- | --- | --- | --- | --- | --- | --- | --- | --- | --- |
| Age | 0.01 | 0.01 | 0.79 | 0.429 | 1.01 (0.99 - 1.02) |  |  |  |  |  |
| Number of Stents | 0.21 | 0.04 | 5.15 | <.001 | 1.23 (1.14 - 1.33) | 0.22 | 0.05 | 4.18 | <.001 | 1.25 (1.12 - 1.38) |
| Total Stent Length | 0.01 | 0.00 | 5.11 | <.001 | 1.01 (1.01 - 1.01) |  |  |  |  |  |
| Sex |  |  |  |  |  |  |  |  |  |  |
| 0 |  |  |  |  | Ref |  |  |  |  | Ref |
| 1 | 0.22 | 0.10 | 2.20 | 0.028 | 1.24 (1.02 - 1.51) | 0.17 | 0.10 | 1.74 | 0.081 | 1.19 (0.98 - 1.45) |
| Glycemic status |  |  |  |  |  |  |  |  |  |  |
| 0 |  |  |  |  | Ref |  |  |  |  | Ref |
| 1 | 0.54 | 0.14 | 3.86 | <.001 | 1.71 (1.30 - 2.25) | 0.50 | 0.14 | 3.51 | <.001 | 1.64 (1.24 - 2.17) |
| 2 | 0.32 | 0.11 | 2.99 | 0.003 | 1.37 (1.12 - 1.69) | 0.22 | 0.11 | 2.01 | 0.044 | 1.24 (1.01 - 1.54) |
| Hypertension |  |  |  |  |  |  |  |  |  |  |
| 1 |  |  |  |  | Ref |  |  |  |  |  |
| 0 | 0.11 | 0.10 | 1.02 | 0.307 | 1.11 (0.91 - 1.36) |  |  |  |  |  |
| Atrial fibrillation |  |  |  |  |  |  |  |  |  |  |
| 0 |  |  |  |  | Ref |  |  |  |  |  |
| 1 | 0.00 | 0.23 | 0.01 | 0.993 | 1.00 (0.64 - 1.57) |  |  |  |  |  |
| Smoking |  |  |  |  |  |  |  |  |  |  |
| 0 |  |  |  |  | Ref |  |  |  |  |  |
| 1 | 0.08 | 0.12 | 0.72 | 0.470 | 1.09 (0.86 - 1.37) |  |  |  |  |  |
| ST segment elevation myocardial infarction |  |  |  |  |  |  |  |  |  |  |
| 0 |  |  |  |  | Ref |  |  |  |  | Ref |
| 1 | 0.39 | 0.11 | 3.42 | <.001 | 1.48 (1.18 - 1.85) | 0.34 | 0.12 | 2.84 | 0.005 | 1.41 (1.11 - 1.78) |
| Cardiogenic shock |  |  |  |  |  |  |  |  |  |  |
| 0 |  |  |  |  | Ref |  |  |  |  | Ref |
| 1 | 1.72 | 0.16 | 10.92 | <.001 | 5.61 (4.12 - 7.64) | 1.51 | 0.17 | 9.08 | <.001 | 4.54 (3.28 - 6.30) |
| Previous MI |  |  |  |  |  |  |  |  |  |  |
| 0 |  |  |  |  | Ref |  |  |  |  |  |
| 1 | 0.46 | 0.58 | 0.79 | 0.427 | 1.58 (0.51 - 4.93) |  |  |  |  |  |
| Previous PCI |  |  |  |  |  |  |  |  |  |  |
| 0 |  |  |  |  | Ref |  |  |  |  | Ref |
| 1 | 0.53 | 0.34 | 1.57 | 0.117 | 1.70 (0.88 - 3.28) | 0.61 | 0.34 | 1.79 | 0.074 | 1.85 (0.94 - 3.63) |
| Previous CABG |  |  |  |  |  |  |  |  |  |  |
| 0 |  |  |  |  | Ref |  |  |  |  |  |
| 1 | -0.63 | 1.00 | -0.63 | 0.528 | 0.53 (0.07 - 3.78) |  |  |  |  |  |
| CKD |  |  |  |  |  |  |  |  |  |  |
| 0 |  |  |  |  | Ref |  |  |  |  | Ref |
| 1 | 0.64 | 0.16 | 3.87 | <.001 | 1.89 (1.37 - 2.61) | 0.54 | 0.17 | 3.20 | 0.001 | 1.71 (1.23 - 2.37) |
| Multi vessel disease |  |  |  |  |  |  |  |  |  |  |
| 0 |  |  |  |  | Ref |  |  |  |  | Ref |
| 1 | 0.27 | 0.10 | 2.75 | 0.006 | 1.32 (1.08 - 1.60) | -0.21 | 0.14 | -1.47 | 0.140 | 0.81 (0.62 - 1.07) |
| Left main coronary lesion |  |  |  |  |  |  |  |  |  |  |
| 0 |  |  |  |  | Ref |  |  |  |  | Ref |
| 1 | 0.63 | 0.18 | 3.49 | <.001 | 1.88 (1.32 - 2.68) | 0.46 | 0.20 | 2.32 | 0.021 | 1.59 (1.07 - 2.35) |
| Bifurcation lesion |  |  |  |  |  |  |  |  |  |  |
| 1 |  |  |  |  | Ref |  |  |  |  | Ref |
| 0 | -0.20 | 0.10 | -2.02 | 0.043 | 0.82 (0.67 - 0.99) | -0.18 | 0.11 | -1.75 | 0.079 | 0.83 (0.68 - 1.02) |
| Calcification lesion |  |  |  |  |  |  |  |  |  |  |
| 0 |  |  |  |  | Ref |  |  |  |  |  |
| 1 | 0.24 | 0.12 | 2.10 | 0.036 | 1.28 (1.02 - 1.60) |  |  |  |  |  |
| Diffuse long lesion |  |  |  |  |  |  |  |  |  |  |
| 0 |  |  |  |  | Ref |  |  |  |  |  |
| 1 | 0.30 | 0.10 | 3.16 | 0.002 | 1.35 (1.12 - 1.63) |  |  |  |  |  |
| Chronic total occlusion |  |  |  |  |  |  |  |  |  |  |
| 0 |  |  |  |  | Ref |  |  |  |  |  |
| 1 | 0.36 | 0.16 | 2.26 | 0.024 | 1.44 (1.05 - 1.97) |  |  |  |  |  |

**Univariate and multivariate Cox analysis for cardiac death**

| **Variables** | **Beta** | **S.E** | **Z** | **P** | **HR (95%CI)** | **m_Beta** | **m_S.E** | **m_Z** | **aP** | **aHR (95%CI)** |
| --- | --- | --- | --- | --- | --- | --- | --- | --- | --- | --- |
| Age | 0.06 | 0.01 | 4.38 | <.001 | 1.06 (1.03 - 1.09) | 0.06 | 0.01 | 4.30 | <.001 | 1.06 (1.03 - 1.09) |
| Number of Stents | 0.23 | 0.07 | 3.12 | 0.002 | 1.25 (1.09 - 1.44) |  |  |  |  |  |
| Total Stent Length | 0.01 | 0.00 | 2.88 | 0.004 | 1.01 (1.01 - 1.01) |  |  |  |  |  |
| Sex |  |  |  |  |  |  |  |  |  |  |
| 0 |  |  |  |  | Ref |  |  |  |  |  |
| 1 | 0.06 | 0.18 | 0.35 | 0.730 | 1.06 (0.75 - 1.52) |  |  |  |  |  |
| Glycemic status |  |  |  |  |  |  |  |  |  |  |
| 0 |  |  |  |  | Ref |  |  |  |  | Ref |
| 1 | 0.71 | 0.26 | 2.74 | 0.006 | 2.03 (1.22 - 3.36) | 0.77 | 0.26 | 2.94 | 0.003 | 2.15 (1.29 - 3.59) |
| 2 | 0.54 | 0.20 | 2.75 | 0.006 | 1.72 (1.17 - 2.52) | 0.36 | 0.20 | 1.83 | 0.068 | 1.44 (0.97 - 2.13) |
| Hypertension |  |  |  |  |  |  |  |  |  |  |
| 1 |  |  |  |  | Ref |  |  |  |  |  |
| 0 | 0.04 | 0.20 | 0.22 | 0.823 | 1.04 (0.71 - 1.53) |  |  |  |  |  |
| Atrial fibrillation |  |  |  |  |  |  |  |  |  |  |
| 0 |  |  |  |  | Ref |  |  |  |  |  |
| 1 | 0.47 | 0.35 | 1.36 | 0.173 | 1.60 (0.81 - 3.16) |  |  |  |  |  |
| Smoking |  |  |  |  |  |  |  |  |  |  |
| 0 |  |  |  |  | Ref |  |  |  |  |  |
| 1 | -0.20 | 0.24 | -0.85 | 0.394 | 0.82 (0.51 - 1.30) |  |  |  |  |  |
| ST segment elevation myocardial infarction |  |  |  |  |  |  |  |  |  |  |
| 0 |  |  |  |  | Ref |  |  |  |  | Ref |
| 1 | 1.09 | 0.18 | 5.93 | <.001 | 2.99 (2.08 - 4.29) | 0.81 | 0.20 | 4.10 | <.001 | 2.25 (1.53 - 3.32) |
| Cardiogenic shock |  |  |  |  |  |  |  |  |  |  |
| 0 |  |  |  |  | Ref |  |  |  |  | Ref |
| 1 | 2.71 | 0.20 | 13.42 | <.001 | 15.08 (10.14 - 22.41) | 2.29 | 0.22 | 10.43 | <.001 | 9.85 (6.41 - 15.14) |
| Previous MI |  |  |  |  |  |  |  |  |  |  |
| 0 |  |  |  |  | Ref |  |  |  |  |  |
| 1 | 0.46 | 1.00 | 0.46 | 0.644 | 1.59 (0.22 - 11.38) |  |  |  |  |  |
| Previous PCI |  |  |  |  |  |  |  |  |  |  |
| 0 |  |  |  |  | Ref |  |  |  |  |  |
| 1 | 0.70 | 0.58 | 1.20 | 0.230 | 2.02 (0.64 - 6.34) |  |  |  |  |  |
| Previous CABG |  |  |  |  |  |  |  |  |  |  |
| 0 |  |  |  |  | Ref |  |  |  |  |  |
| 1 | -14.01 | 1500.00 | -0.01 | 0.993 | 0.00 (0.00 - Inf) |  |  |  |  |  |
| CKD |  |  |  |  |  |  |  |  |  |  |
| 0 |  |  |  |  | Ref |  |  |  |  | Ref |
| 1 | 1.20 | 0.24 | 4.91 | <.001 | 3.32 (2.06 - 5.37) | 1.18 | 0.25 | 4.72 | <.001 | 3.24 (1.99 - 5.28) |
| Multi vessel disease |  |  |  |  |  |  |  |  |  |  |
| 0 |  |  |  |  | Ref |  |  |  |  |  |
| 1 | 0.45 | 0.18 | 2.50 | 0.013 | 1.57 (1.10 - 2.25) |  |  |  |  |  |
| Left main coronary lesion |  |  |  |  |  |  |  |  |  |  |
| 0 |  |  |  |  | Ref |  |  |  |  | Ref |
| 1 | 1.30 | 0.25 | 5.12 | <.001 | 3.68 (2.24 - 6.07) | 0.96 | 0.26 | 3.65 | <.001 | 2.60 (1.56 - 4.34) |
| Bifurcation lesion |  |  |  |  |  |  |  |  |  |  |
| 1 |  |  |  |  | Ref |  |  |  |  |  |
| 0 | -0.19 | 0.19 | -1.01 | 0.313 | 0.83 (0.57 - 1.19) |  |  |  |  |  |
| Calcification lesion |  |  |  |  |  |  |  |  |  |  |
| 0 |  |  |  |  | Ref |  |  |  |  |  |
| 1 | 0.46 | 0.20 | 2.29 | 0.022 | 1.59 (1.07 - 2.37) |  |  |  |  |  |
| Diffuse long lesion |  |  |  |  |  |  |  |  |  |  |
| 0 |  |  |  |  | Ref |  |  |  |  |  |
| 1 | 0.18 | 0.18 | 0.99 | 0.324 | 1.19 (0.84 - 1.69) |  |  |  |  |  |
| Chronic total occlusion |  |  |  |  |  |  |  |  |  |  |
| 0 |  |  |  |  | Ref |  |  |  |  |  |
| 1 | 0.08 | 0.33 | 0.24 | 0.814 | 1.08 (0.57 - 2.06) |  |  |  |  |  |

**Univariate and multivariate Cox analysis for repeat revascularization**

| **Variables** | **Beta** | **S.E** | **Z** | **P** | **HR (95%CI)** | **m_Beta** | **m_S.E** | **m_Z** | **aP** | **aHR (95%CI)** |
| --- | --- | --- | --- | --- | --- | --- | --- | --- | --- | --- |
| Age | -0.02 | 0.01 | -2.16 | 0.031 | 0.98 (0.96 - 0.99) | -0.02 | 0.01 | -1.62 | 0.106 | 0.98 (0.96 - 1.00) |
| Number of Stents | 0.18 | 0.05 | 3.68 | <.001 | 1.19 (1.09 - 1.31) | 0.17 | 0.05 | 3.50 | <.001 | 1.19 (1.08 - 1.30) |
| Total Stent Length | 0.01 | 0.00 | 3.57 | <.001 | 1.01 (1.01 - 1.01) |  |  |  |  |  |
| Sex |  |  |  |  |  |  |  |  |  |  |
| 0 |  |  |  |  | Ref |  |  |  |  | Ref |
| 1 | 0.30 | 0.12 | 2.59 | 0.010 | 1.35 (1.08 - 1.69) | 0.25 | 0.12 | 2.11 | 0.035 | 1.28 (1.02 - 1.62) |
| Glycemic status |  |  |  |  |  |  |  |  |  |  |
| 0 |  |  |  |  | Ref |  |  |  |  | Ref |
| 1 | 0.40 | 0.17 | 2.39 | 0.017 | 1.49 (1.07 - 2.07) | 0.42 | 0.17 | 2.47 | 0.013 | 1.52 (1.09 - 2.11) |
| 2 | 0.26 | 0.12 | 2.10 | 0.036 | 1.29 (1.02 - 1.64) | 0.22 | 0.13 | 1.74 | 0.082 | 1.24 (0.97 - 1.59) |
| Hypertension |  |  |  |  |  |  |  |  |  |  |
| 1 |  |  |  |  | Ref |  |  |  |  | Ref |
| 0 | 0.17 | 0.12 | 1.43 | 0.151 | 1.19 (0.94 - 1.50) | 0.18 | 0.12 | 1.47 | 0.143 | 1.20 (0.94 - 1.52) |
| Atrial fibrillation |  |  |  |  |  |  |  |  |  |  |
| 0 |  |  |  |  | Ref |  |  |  |  |  |
| 1 | -0.30 | 0.31 | -0.97 | 0.334 | 0.74 (0.41 - 1.36) |  |  |  |  |  |
| Smoking |  |  |  |  |  |  |  |  |  |  |
| 0 |  |  |  |  | Ref |  |  |  |  |  |
| 1 | 0.19 | 0.13 | 1.43 | 0.154 | 1.21 (0.93 - 1.57) |  |  |  |  |  |
| ST segment elevation myocardial infarction |  |  |  |  |  |  |  |  |  |  |
| 0 |  |  |  |  | Ref |  |  |  |  |  |
| 1 | 0.07 | 0.15 | 0.51 | 0.613 | 1.08 (0.81 - 1.44) |  |  |  |  |  |
| Cardiogenic shock |  |  |  |  |  |  |  |  |  |  |
| 0 |  |  |  |  | Ref |  |  |  |  | Ref |
| 1 | 0.76 | 0.28 | 2.68 | 0.007 | 2.14 (1.23 - 3.72) | 0.63 | 0.29 | 2.21 | 0.027 | 1.88 (1.07 - 3.29) |
| Previous MI |  |  |  |  |  |  |  |  |  |  |
| 0 |  |  |  |  | Ref |  |  |  |  |  |
| 1 | 0.39 | 0.71 | 0.55 | 0.585 | 1.47 (0.37 - 5.91) |  |  |  |  |  |
| Previous PCI |  |  |  |  |  |  |  |  |  |  |
| 0 |  |  |  |  | Ref |  |  |  |  |  |
| 1 | 0.41 | 0.41 | 1.00 | 0.315 | 1.51 (0.67 - 3.39) |  |  |  |  |  |
| Previous CABG |  |  |  |  |  |  |  |  |  |  |
| 0 |  |  |  |  | Ref |  |  |  |  | Ref |
| 1 | -14.01 | 907.77 | -0.02 | 0.988 | 0.00 (0.00 - Inf) | -14.03 | 923.91 | -0.02 | 0.988 | 0.00 (0.00 - Inf) |
| CKD |  |  |  |  |  |  |  |  |  |  |
| 0 |  |  |  |  | Ref |  |  |  |  | Ref |
| 1 | 0.40 | 0.21 | 1.87 | 0.062 | 1.49 (0.98 - 2.25) | 0.32 | 0.22 | 1.50 | 0.133 | 1.38 (0.91 - 2.11) |
| Multi vessel disease |  |  |  |  |  |  |  |  |  |  |
| 0 |  |  |  |  | Ref |  |  |  |  |  |
| 1 | 0.19 | 0.12 | 1.59 | 0.111 | 1.21 (0.96 - 1.52) |  |  |  |  |  |
| Left main coronary lesion |  |  |  |  |  |  |  |  |  |  |
| 0 |  |  |  |  | Ref |  |  |  |  |  |
| 1 | 0.04 | 0.27 | 0.13 | 0.894 | 1.04 (0.61 - 1.77) |  |  |  |  |  |
| Bifurcation lesion |  |  |  |  |  |  |  |  |  |  |
| 1 |  |  |  |  | Ref |  |  |  |  |  |
| 0 | -0.16 | 0.12 | -1.37 | 0.170 | 0.85 (0.68 - 1.07) |  |  |  |  |  |
| Calcification lesion |  |  |  |  |  |  |  |  |  |  |
| 0 |  |  |  |  | Ref |  |  |  |  |  |
| 1 | 0.15 | 0.14 | 1.05 | 0.294 | 1.16 (0.88 - 1.52) |  |  |  |  |  |
| Diffuse long lesion |  |  |  |  |  |  |  |  |  |  |
| 0 |  |  |  |  | Ref |  |  |  |  |  |
| 1 | 0.30 | 0.11 | 2.68 | 0.007 | 1.35 (1.08 - 1.68) |  |  |  |  |  |
| Chronic total occlusion |  |  |  |  |  |  |  |  |  |  |
| 0 |  |  |  |  | Ref |  |  |  |  |  |
| 1 | 0.44 | 0.18 | 2.43 | 0.015 | 1.55 (1.09 - 2.22) |  |  |  |  |  |

**Univariate and multivariate Cox analysis for non-fatal myocardial infarction**

| **Variables** | **Beta** | **S.E** | **Z** | **P** | **HR (95%CI)** | **m_Beta** | **m_S.E** | **m_Z** | **aP** | **aHR (95%CI)** |
| --- | --- | --- | --- | --- | --- | --- | --- | --- | --- | --- |
| Age | 0.04 | 0.02 | 2.10 | 0.035 | 1.04 (1.01 - 1.07) | 0.04 | 0.02 | 2.11 | 0.035 | 1.04 (1.01 - 1.07) |
| Number of Stents | 0.15 | 0.09 | 1.58 | 0.114 | 1.16 (0.97 - 1.39) |  |  |  |  |  |
| Total Stent Length | 0.00 | 0.00 | 1.24 | 0.216 | 1.00 (1.00 - 1.01) |  |  |  |  |  |
| Sex |  |  |  |  |  |  |  |  |  |  |
| 0 |  |  |  |  | Ref |  |  |  |  |  |
| 1 | -0.05 | 0.22 | -0.25 | 0.801 | 0.95 (0.62 - 1.45) |  |  |  |  |  |
| Glycemic status |  |  |  |  |  |  |  |  |  |  |
| 0 |  |  |  |  | Ref |  |  |  |  | Ref |
| 1 | 0.52 | 0.29 | 1.81 | 0.071 | 1.68 (0.96 - 2.96) | 0.51 | 0.29 | 1.74 | 0.081 | 1.66 (0.94 - 2.92) |
| 2 | -0.19 | 0.26 | -0.73 | 0.468 | 0.83 (0.50 - 1.37) | -0.29 | 0.26 | -1.10 | 0.271 | 0.75 (0.45 - 1.25) |
| Hypertension |  |  |  |  |  |  |  |  |  |  |
| 1 |  |  |  |  | Ref |  |  |  |  |  |
| 0 | 0.11 | 0.23 | 0.49 | 0.621 | 1.12 (0.71 - 1.77) |  |  |  |  |  |
| Atrial fibrillation |  |  |  |  |  |  |  |  |  |  |
| 0 |  |  |  |  | Ref |  |  |  |  |  |
| 1 | 0.27 | 0.46 | 0.58 | 0.564 | 1.30 (0.53 - 3.22) |  |  |  |  |  |
| Smoking |  |  |  |  |  |  |  |  |  |  |
| 0 |  |  |  |  | Ref |  |  |  |  |  |
| 1 | -0.17 | 0.28 | -0.59 | 0.554 | 0.85 (0.48 - 1.47) |  |  |  |  |  |
| ST segment elevation myocardial infarction |  |  |  |  |  |  |  |  |  |  |
| 0 |  |  |  |  | Ref |  |  |  |  |  |
| 1 | 0.40 | 0.25 | 1.58 | 0.115 | 1.49 (0.91 - 2.46) |  |  |  |  |  |
| Cardiogenic shock |  |  |  |  |  |  |  |  |  |  |
| 0 |  |  |  |  | Ref |  |  |  |  | Ref |
| 1 | 1.30 | 0.40 | 3.30 | <.001 | 3.69 (1.70 - 8.00) | 1.30 | 0.40 | 3.27 | 0.001 | 3.66 (1.68 - 7.97) |
| Previous MI |  |  |  |  |  |  |  |  |  |  |
| 0 |  |  |  |  | Ref |  |  |  |  |  |
| 1 | -14.01 | 1666.27 | -0.01 | 0.993 | 0.00 (0.00 - Inf) |  |  |  |  |  |
| Previous PCI |  |  |  |  |  |  |  |  |  |  |
| 0 |  |  |  |  | Ref |  |  |  |  |  |
| 1 | 1.07 | 0.59 | 1.83 | 0.068 | 2.92 (0.92 - 9.25) |  |  |  |  |  |
| Previous CABG |  |  |  |  |  |  |  |  |  |  |
| 0 |  |  |  |  | Ref |  |  |  |  |  |
| 1 | 1.07 | 1.01 | 1.07 | 0.286 | 2.93 (0.41 - 21.03) |  |  |  |  |  |
| CKD |  |  |  |  |  |  |  |  |  |  |
| 0 |  |  |  |  | Ref |  |  |  |  | Ref |
| 1 | 0.78 | 0.35 | 2.20 | 0.028 | 2.17 (1.09 - 4.33) | 0.81 | 0.36 | 2.26 | 0.024 | 2.24 (1.11 - 4.51) |
| Multi vessel disease |  |  |  |  |  |  |  |  |  |  |
| 0 |  |  |  |  | Ref |  |  |  |  |  |
| 1 | 0.30 | 0.22 | 1.34 | 0.180 | 1.35 (0.87 - 2.08) |  |  |  |  |  |
| Left main coronary lesion |  |  |  |  |  |  |  |  |  |  |
| 0 |  |  |  |  | Ref |  |  |  |  |  |
| 1 | 0.67 | 0.39 | 1.71 | 0.088 | 1.96 (0.91 - 4.24) |  |  |  |  |  |
| Bifurcation lesion |  |  |  |  |  |  |  |  |  |  |
| 1 |  |  |  |  | Ref |  |  |  |  |  |
| 0 | -0.17 | 0.22 | -0.76 | 0.450 | 0.84 (0.54 - 1.31) |  |  |  |  |  |
| Calcification lesion |  |  |  |  |  |  |  |  |  |  |
| 0 |  |  |  |  | Ref |  |  |  |  |  |
| 1 | 0.09 | 0.27 | 0.33 | 0.743 | 1.09 (0.64 - 1.86) |  |  |  |  |  |
| Diffuse long lesion |  |  |  |  |  |  |  |  |  |  |
| 0 |  |  |  |  | Ref |  |  |  |  |  |
| 1 | 0.07 | 0.22 | 0.34 | 0.730 | 1.08 (0.71 - 1.64) |  |  |  |  |  |
| Chronic total occlusion |  |  |  |  |  |  |  |  |  |  |
| 0 |  |  |  |  | Ref |  |  |  |  |  |
| 1 | -0.08 | 0.42 | -0.19 | 0.852 | 0.92 (0.40 - 2.12) |  |  |  |  |  |
